# Self-Shielding Gyroscopic Radiosurgery: A Prospective Experience and Analysis of the First 100 Patients

**DOI:** 10.7759/cureus.56035

**Published:** 2024-03-12

**Authors:** Felix Ehret, Nadja Kohlhase, Dochka Eftimova, Theresa Hofmann, Christoph Fürweger, Alfred Haidenberger, Markus Kufeld, Alexander Muacevic, Antonio Santacroce

**Affiliations:** 1 Radiation Oncology, Charité – Universitätsmedizin Berlin, Corporate Member of Freie Universität Berlin and Humboldt-Universität zu Berlin, Berlin, DEU; 2 Charité – Universitätsmedizin Berlin, German Cancer Consortium (DKTK) partner site Berlin, and German Cancer Research Center (DKFZ) Heidelberg, Berlin, DEU; 3 Radiation Oncology, European Radiosurgery Center Munich, Munich, DEU; 4 Medical Physics, European Radiosurgery Center Munich, Munich, DEU; 5 Stereotaxy and Functional Neurosurgery, Center for Neurosurgery, University Hospital Cologne, Cologne, DEU; 6 Radiosurgery, European Radiosurgery Center Munich, Munich, DEU; 7 Medicine, Faculty of Health, Witten/Herdecke University, Witten, DEU; 8 Neurosurgery, St. Barbara-Klinik Hamm-Heessen, Hamm, DEU

**Keywords:** radiosurgery, meningioma, vestibular schwannoma, brain metastasis, neurosurgery, radiation oncology, stereotactic radiosurgery

## Abstract

Background

Stereotactic radiosurgery is a well-established treatment option for the management of various benign and malignant brain tumors. It can be delivered with several treatment platforms, usually requiring shielded radiation vaults to meet regulatory safety requirements. Recent technical advances have led to the first self-shielding platform enabling the delivery of gyroscopic radiosurgery (GRS). Given the limited number of GRS treatment platforms, the novelty of its characteristics, and the lack of available data, we report our prospective experience with the first 100 patients treated with GRS.

Materials and methods

Patients undergoing GRS for the treatment of intracranial tumors were enrolled in this prospective study. Patient and treatment characteristics, including patient satisfaction, were collected and analyzed.

Results

A total of 100 patients with 155 tumors were treated. The most commonly treated tumors comprised brain metastases (BM) (49%), vestibular schwannomas (31%), and meningiomas (14%). The median prescription dose for malignant and benign tumors was 20 and 13 Gy, respectively. The median prescription isodose line was 56%. Gross tumor volumes were small, with a median of 0.37 cc for BM and 0.92 cc for the other entities. The median total treatment time was 40 minutes. Dosimetric performance indices showed median values of 1.20 (conformity index), 1.24 (new conformity index), 1.74 (homogeneity index), and 3.13 (gradient index). Volumetric assessment of the treated tumors showed an overall decrease in size at the first available follow-up. Most patients were satisfied with the treatment experience.

Conclusion

Our first prospective experience of the use of GRS is favorable. Analyses of the dosimetric performance, treatment times, volumetric assessment, and patient satisfaction demonstrate its suitability for stereotactic treatments of intracranial tumors. Further prospective clinical and dosimetric analyses for GRS are pending.

## Introduction

Stereotactic radiosurgery (SRS) is a well-established treatment option for the management of various benign and malignant brain tumors [1,2]. Available evidence of its efficacy and safety has been demonstrated with the treatment of brain metastases (BM), meningiomas, arteriovenous malformations, vestibular schwannomas, paragangliomas, and pituitary adenomas [2,3]. Radiosurgery plays a particularly important role in the management of BM nowadays, given the paradigm shift from whole brain radiotherapy (WBRT) toward metastasis-directed treatments. SRS can be delivered with a variety of treatment platforms, including the Gamma Knife, CyberKnife, and other dedicated stereotactic linear accelerators. In order to avoid exposure of persons other than the patients and to comply with regulatory safety requirements, treatment vaults are usually required to deliver not only SRS treatments but also radiation therapy in general. However, the spatial conditions and financial resources to construct such a vault are not universally available. Recent technical advances have led to the first self-shielding platform allowing the delivery of gyroscopic radiosurgery (GRS) [4-7]. This enables radiation oncologists and neurosurgeons to deliver SRS treatments without a vault, potentially increasing the availability of such therapies in areas where shielded vaults cannot be built. Global access to radiotherapy, in general, and radiosurgery, in particular, is highly heterogeneous, given the demand for qualified personnel, radiation vaults, and treatment tools [8-12]. As technical advances have always played a fundamental role in radiation oncology and radiosurgery, the dedicated analysis of treatment platforms is crucial to assess their effectiveness, safety, and performance in the daily clinical routine [8,13]. Given the current paucity of available GRS treatment platforms, the novelty of its characteristics, and the lack of data, we report our experience of the first 100 patients treated with GRS in the setting of a prospective study as an example of how to scientifically evaluate the introduction of new technology in the field of radiation oncology and radiosurgery from the very beginning.

## Materials and methods

Patients enrolled in the prospective study entitled ‘Self-Shielding Gyroscopic Radiosurgery - a First Prospective Observational Study and Retrospective Comparison’ (GRAY I) (clinical trials identifier: DRKS00025820), treated between December 2021 and November 2022 for a benign or malignant intracranial tumor with GRS and at least one available clinical and radiographic follow-up until March 2023 were included in this analysis. All treatments were delivered with the ZAP-X® (ZAP Surgical Systems Inc., San Carlos, CA, USA) GRS platform in a single session. Treatment planning was performed utilizing the ZAP-X® treatment planning system (Version 1.8.55 - 1.8.58) and prior imaging with computed tomography and contrast-enhanced magnetic resonance imaging. The planning target volume (PTV) was created by adding the smallest feasible margin, i.e., an isotropic margin of one CT voxel (0.5 - 1 mm) to the gross tumor volume (GTV), which was cut to 0 mm toward adjacent organs at risk. For pre-treatment plan verification, selected plans were verified by independent, Monte-Carlo-based secondary dose calculations using SciMoCa (Scientific RT, Munich, Germany). In addition, real-time verification was performed by measuring beams exiting the patient with an integrated MV assessment. Treatment immobilization was achieved with a non-invasive thermoplastic mask. Intrafraction head motion was compensated by the acquisition of planar kV images from different angles in 45-second intervals and registration of each image to a digitally reconstructed radiograph, with immediate correction of the head position by moving the couch. Patients with malignant tumors underwent the first follow-up three months after treatment. For benign lesions, the first follow-up was scheduled six months after GRS. Toxicity was graded according to the Common Terminology Criteria for Adverse Events (CTCAE), version 5.0. Patient satisfaction with the treatment experience was optionally measured during the first follow-up on a five-level Likert scale (“1” to “5”, “1” stands for “very satisfied” and “5” for “very disappointed”). The conformity index (CI, based on the BrainSCAN definition), new CI (nCI), homogeneity index (HI), and dose gradient index (GI) were assessed and calculated as previously described [14-16]. Tumor and treatment characteristics were collected and stored. Treatment times were defined as follows: total treatment time is the time to complete the whole treatment, setup time is the period required to complete the kV image-guided alignment of the head, and the delivery time is defined as the time duration between the user pressing the start button or resume the treatment and the last beam state change, i.e., beam off. This also includes periodic acquisition and review of kV images during treatment and any interruptions due to safety interlocks, e.g., system components moving close to the patient. All treatment targets underwent volumetric assessment at their first follow-up. For patients with multiple BM, the volume of metastases was summarized. Two patients developed extensive leptomeningeal disease after SRS, their volumetric data were excluded from the analysis. Each benign tumor was counted separately. Volumetric data were analyzed utilizing a Wilcoxon sign-rank test. Assessment for correlation was done using Spearman's rank correlation coefficient ρ. p-values ≤ 0.05 were considered significant. Statistical analysis was performed using STATA MP 17.0 (StataCorp, College Station, TX, USA). Figures were created with STATA MP 17.0 and GraphPad Prism 8.01 (GraphPad Software, San Diego, CA, USA). All study participants provided informed consent prior to study enrollment and treatment. This study was approved by the local institutional review board.

## Results

**Figure 1:**
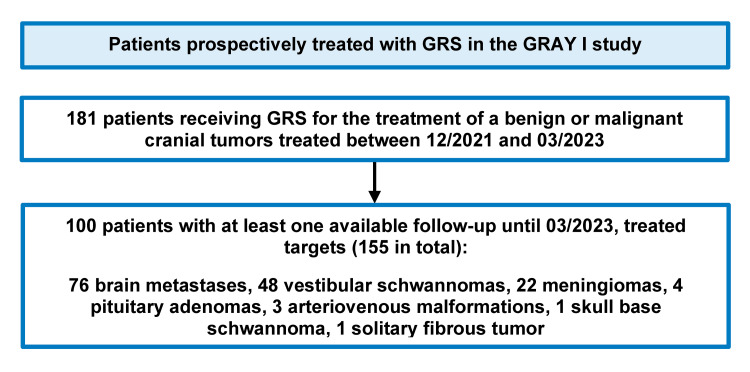
Details of enrolled and analyzed patients with treatment targets. GRS: Gyroscopic radiosurgery

A total of 181 patients were enrolled between December 2021 and March 2023. One hundred patients, 42 men and 58 women, with at least one available follow-up, and a total of 155 targets were included in this analysis. The included patients and targets are shown in Figure 1.

**Figure 2:**
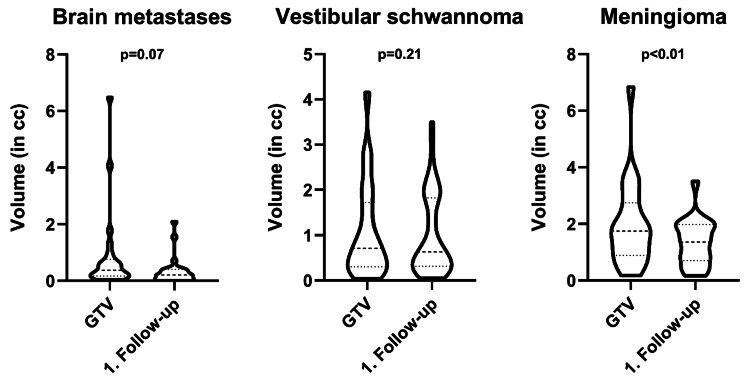
Volumetry of brain metastases, vestibular schwannomas, and meningiomas at treatment and first follow-up. cc: Cubic centimeters, GTV: gross tumor volume. Dashed line: median, dotted lines: 25th-75th interquartile range

The most commonly treated tumors were BM (n = 76, 49%), vestibular schwannomas (n = 48, 31%), and meningiomas (n = 22, 14%, presumed grading per imaging or pathology: 18 grade 1, 4 grade 2 tumors). The median age at treatment was 56.8 years. The ratio of BM to benign tumors was 1:1.03. Most patients were treated for a single target (n = 80, 80%), with a maximum of 10 targets in two patients with multiple BM. The median prescription dose for malignant (BM) and benign tumors was 20 and 13 Gy, respectively. The corresponding median prescription isodose lines were 58 and 56%. The median GTV of BM was 0.37 cc and 0.92 cc for the other entities. Dosimetric performance indices showed median values of 1.20 (CI), 1.24 (nCI), 1.74 (HI), and 3.13 (GI). The median coverage was 98.4%. Volumetric assessment of the treated tumors showed an overall decrease in volume at the first available follow-up (all tumors p < 0.01, Figure 2).

**Figure 3:**
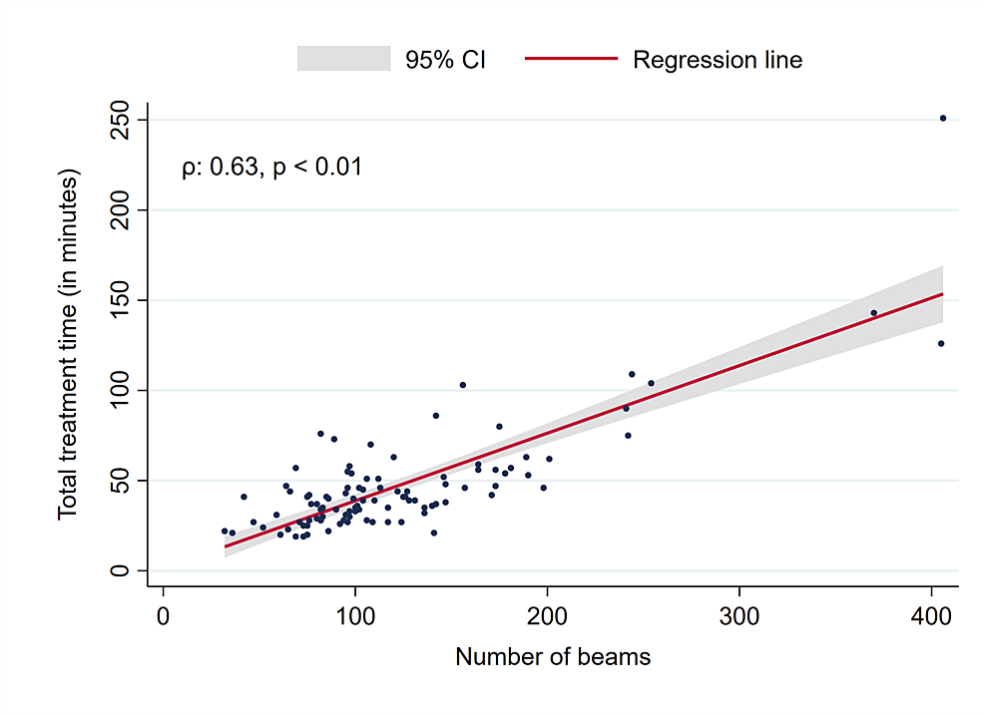
Total treatment time vs. the number of beams. CI: Confidence interval.

**Figure 4:**
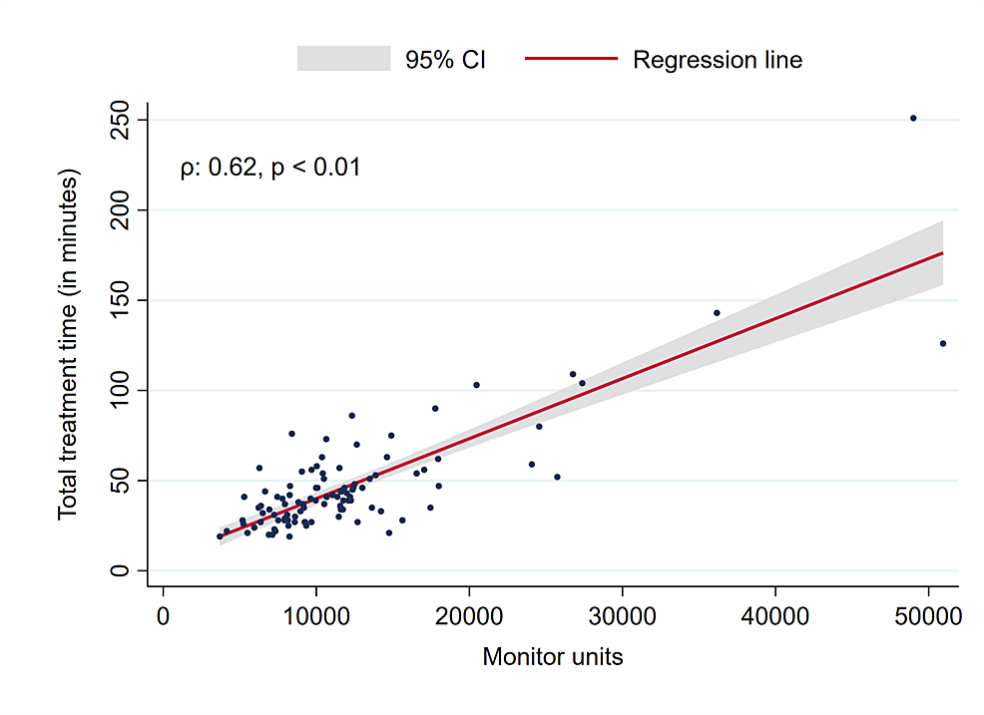
Total treatment time vs. monitor units. CI: Confidence interval.

While meningiomas showed a significant reduction in volume at the first follow-up, BM and vestibular schwannomas did not, the latter having a proportion of tumors (22/48, 45.8%) with swelling, i.e., volume increase (Figure 2). Two patients with BM developed extensive leptomeningeal disease after SRS. Recorded adverse events grade ≥3 comprised two grade 3 vertigo (grade 3 and grade 2 before SRS), one grade 3 tinnitus (grade 2 before treatment), and one new grade 3 facial nerve disorder/palsy, which was not present before GRS. The facial nerve disorder was rated House-Brackmann grade V at the first follow-up after six months and improved to grade IV after a year. One patient had grade 3 hearing impairment before treatment which remained stable at the first follow-up (grade 3). A total of 82 patients agreed to report their overall treatment experience with GRS (response rate 82%). The vast majority of patients were “very satisfied” (75 patients, 91.4%), six patients (7.3%) selected “2” on the scale, while only one patient was disappointed (“5”). The median total treatment time was 40 minutes, which increased with the number of beams, monitor units, isocenters, and PTV size (Figures 3, 4, data for the number of isocenters and PTV not shown, all p < 0.01).

Most treatments were completed in an hour or less (n = 84, 84%). In three cases, treatments took longer than 120 minutes due to the number of targets (two patients with 10 BM, one with 6 BM) and, in two cases, due to a prolonged setup time and breaks during the treatment. Patient and treatment characteristics are summarized in Table 1.

**Table 1:** Patient, tumor, and treatment characteristics. SD: Standard deviation, IQR: 25%-75% interquartile range GTV: gross tumor volume, PTV: planning target volume, cc: cubic centimeters, min: minutes.

Number of patients	100			
Number of targets	155			
Sex (male/female, %)	42/58			
	Median	Mean (SD)	IQR	Range
Age (years)	56.8	56.4	48.9 – 64.5	23.8 – 85.2
Number of treated lesions per treatment	1	1.55 (1.57)	1 – 1	1 – 10
Prescription dose benign tumors (Gy)	13	13.9 (1.57)	13 – 15	13 – 20
Prescription isodose line benign tumors (%)	56	55.4 (5.4)	52 – 58	43.7 – 72
GTV benign tumors (cc)	0.92	1.42 (1.32)	0.38 – 2.07	0.04 – 6.84
PTV benign tumors (cc)	1.32	2.01 (1.80)	0.58 – 2.69	0.09 – 8.50
Prescription dose malignant tumors (Gy)	20	20 (0.95)	20 – 21	18 – 22
Prescription isodose line malignant tumors (%)	58	58.8 (8.17)	52 – 62	49 – 82.4
GTV malignant tumors (cc)	0.37	0.86 (1.46)	0.18 – 0.72	0.04 – 6.48
PTV malignant tumors (cc)	0.69	1.67 (2.43)	0.34 – 1.53	0.04 – 8.93
Conformity index	1.20	1.26 (0.20)	1.15 – 1.27	1.09 – 2.17
Normal conformity index	1.24	1.28 (0.16)	1.19 – 1.29	1.13 – 2.34
Homogeneity index	1.74	1.69 (0.24)	1.56 – 1.85	1.18 – 2.22
Gradient index	3.13	3.20 (0.44)	2.88 – 3.41	2.40 – 5.07
Coverage (%)	98.4	97.8 (1.92)	96.7 – 99.1	92.1 – 100
Total treatment time (min)	40	47 (30.6)	29.5 – 53.5	19 – 197
Setup time (min)	5.7	9.7 (10.0)	2.6 – 12.3	2.4 – 46.4
Delivery time (min)	29.9	35.2 (25.5)	22.7 – 39.2	7.6 – 165
Number of isocenter	8	9.0 (5.2)	6 – 11	1 – 30
Monitor units	10398	12116 (7615)	8025 – 12671	3926 – 49974
Number of beams	102	121.9 (66.5)	82 – 142	34 – 405

## Discussion

While preliminary, retrospective data are available, this study, to our knowledge, represents the first prospective analysis of the use of GRS [6,7]. Adopting new treatment platforms and techniques in the field of radiation oncology is always a challenge for institutions and their personnel but is undoubtedly necessary to achieve measurable progress [17,18]. We decided to assess the efficacy and safety of GRS right from the start in the setting of a prospective clinical study and had a favorable experience thus far. While encountering only a few situations with prolonged setup times, the treatment delivery and results at the first follow-up are sound. Volumetric assessment of treated tumors demonstrated a widespread regression of lesions at the first follow-up, with the expected exception of vestibular schwannomas, which demonstrated partial swelling in selected patients, potentially causing some of the observed grade ≥3 toxicities [19]. In terms of dosimetry, GRS demonstrated a solid performance as indicated by the CI, nCI, HI, and GI measured. Recently, Paddick et al. reported on a benchmarking test on current state-of-the-art SRS platforms, including GRS, CyberKnife, Gamma Knife, Elekta Versa, and Varian TrueBeam, as well as Edge [20]. The study analyzed seven different SRS cases and found comparable results regarding GRS. The coverage was 96.2 and 100% for benign tumors and BM, respectively, which matches well with our median of 98.4%. The GI was also fairly in line with our results (2.74 vs. 3.12 for benign tumors, 3.83 vs. 3.21 for BM) [20]. However, minor deviations may be explained by the considerable difference in analyzed cases (7 vs. 100) and their specific details. While dosimetry is crucial for the efficacy and safety of SRS treatments, and improvements in SRS platforms over time have been reported, patient satisfaction is of utmost importance as well [20-22].

Herein, the treatment experience for the patients was good, as highlighted by the high rate of satisfied study participants, even though not all patients decided to report. Notably, the vast majority of patients treated at our institution decided to enroll in this study (>95% of patients treated with GRS), underlining the general willingness of patients to participate in clinical studies investigating new techniques and treatment tools. Yet, one has to note that this study is non-interventional, which may affect the reported enrollment rates as no experimental treatments were done. Scientific advancements in radiation oncology necessitate the continuous evaluation and improvement of treatment modalities. The introduction of any new technology, such as GRS, must be accompanied by rigorous scientific investigation to ensure its safety, efficacy, and clinical relevance [8,23]. It is imperative to conduct prospective studies, like the present one, to assess the feasibility, dosimetric accuracy, and clinical outcomes associated with new treatment modalities. By doing so, we can establish solid evidence for treatment platforms and facilitate their integration into routine clinical practice. Moreover, continuous and structured reporting will provide further insights concerning incremental changes over time, which may be missed in the absence of a clinical study.

Given the central role SRS has occupied in the management of various benign and malignant intracranial tumors, its availability, as with radiotherapy in general, remains limited on a global scale [8-10,24,25]. One of the primary barriers to the adoption of SRS worldwide is the requirement for specialized radiation vaults equipped with dedicated treatment tools such as linear accelerator-based platforms or Gamma Knife units [8]. These shielded radiation vaults are costly to construct, maintain, and operate, making them inaccessible to many healthcare facilities, particularly in resource-limited settings or challenging spatial surroundings [23,26]. Consequently, a substantial proportion of patients who could potentially benefit from SRS do not have access to this advanced treatment option. This is of great relevance for patients suffering from BM. Throughout recent years, growing evidence has started a paradigm shift toward the stereotactic treatment of a limited number of BM - instead of WBRT - to preserve the cognitive function and quality of life of affected patients [27]. Implementing technological advances that may reduce the barrier to SRS is therefore essential to providing the current standard of care to as many patients as possible. This is particularly important for low- and middle-income countries [28,29]. Pannullo et al. highlighted the need for further advances in the field of SRS, stating “A radiosurgical platform that is inexpensive to install, requires a moderate amount of training to use, and that can be supported remotely could result in significant benefit in improving care of complex neurosurgical conditions without the costs and unknowns of traditional open surgery” [8]. While self-shielding may alleviate financial and constructional challenges in specific settings, further issues in the implementation of SRS remain, such as the demand for trained personnel, energy consumption, and, in the case of Gamma Knife-based SRS, management of radioactive isotopes [8,23,30]. Self-shielding GRS also offers an isotope-free alternative to the well-established, cobalt-based Gamma Knife. However, further efforts are necessary to increase the worldwide availability of SRS.

It is essential to acknowledge that this study represents an initial exploration of GRS with several limitations, and further research is warranted to validate our findings and address several important matters. First, long-term follow-up is needed to evaluate the oncological outcomes of GRS-treated patients. Comprehensive studies between GRS and other SRS platforms, including dosimetric analyses, should also be conducted to provide further insights and optimize treatments and their delivery [20-22]. Additionally, future cost-effectiveness analyses are essential to determine the economic viability of implementing GRS systems in different healthcare settings. In conclusion, the lack of availability of SRS on a global scale is a significant challenge in stereotactic radiation therapy [8]. The emergence of GRS as a potentially viable alternative to traditional SRS techniques offers a promising solution to overcome some of the existing barriers. By eliminating the need for radiation vaults, GRS could have the potential to increase the accessibility of SRS in selected situations and extend its benefits to a larger patient population [8,23]. This versatility holds promise for expanding access to SRS in underserved regions and reducing healthcare disparities associated with advanced radiation therapy [29]. Further studies and collaborative efforts are necessary to fully explore the clinical potential and practical implications of GRS. Prospective analyses of new technologies in radiation oncology are crucial to comprehensively assess performance and safety. We encourage the implementation of comparable studies whenever new tools in the field become available and will report our future experiences with GRS and further in-depth analyses.

## Conclusions

Our first experience of the use of GRS in the setting of a prospective clinical study is favorable with high patient enrollment rates and widespread patient satisfaction. Analyses of the dosimetric performance and treatment times demonstrate its suitability for the stereotactic treatment of intracranial tumors despite the common challenges in the early adoption of new treatment platforms. Given its self-shielding capabilities, the implementation and use of GRS may offer a way to match the increasing demand for SRS. Consistent and continuous assessment of the use of new treatment platforms in radiation oncology is crucial to maintain quality standards and refine future treatments.
